# Supplementary material for: Organic fertilizer type and dose affect growth, morphological and physiological parameters, and mineral nutrition of watermelon seedlings
Source: PeerJ. 2024 Feb 21;12:e16902. doi: 10.7717/peerj.16902 (PMC10893865; doi:10.7717/peerj.16902)
Supplement: Supplemental Information 2 [file peerj-12-16902-s002.docx]

**Table S1**. Inspection reports of BM2 and OM2 substrates.

|  | pH | Particle size | Water | NO_3_ | NH_4_ | Total N | P | K | Ca | Mg | Na | Fe | Cu | Mn | Zn |
| --- | --- | --- | --- | --- | --- | --- | --- | --- | --- | --- | --- | --- | --- | --- | --- |
| unit |  | 10 mesh% | % | mg/kg | mg/kg | mg/kg | mg/kg | mg/kg | mg/kg | mg/kg | mg/kg | mg/kg | mg/kg | mg/kg | mg/kg |
| BM2 | 5.45 | 12.0 | 54.4 | 57.05 | 6.4 | 17.86 | 9.4 | 62.05 | 93.9 | 45.3 | 18.2 | 0.99 | 0.075 | 0.565 | 0.07 |
| OM2 | 5.34 | 12.4 | 54.1 | < 0.6 | < 0.25 | < 0.33 | < 0.21 | 2 | 2.8 | 1.9 | 7.9 | 0.505 | < 0.045 | < 0.03 | < 0.03 |

Data provided by the manufacturer.

**Table S2**. Mineral nutrient contents in fertilizers.

|  | N | P | K | Ca | Mg | Na | S | Fe | Cu | Mn | Zn | B |
| --- | --- | --- | --- | --- | --- | --- | --- | --- | --- | --- | --- | --- |
| unit | % | % | % | % | % | % | % | mg/kg | mg/kg | mg/kg | mg/kg | mg/kg |
| Conventional | 20 | 8.73 | 16.56 | N/A | 0.05 | N/A | N/A | 500 | 125 | 250 | 250 | N/A |
| Sustane | 4 | 2.62 | 3.31 | 4.43 | 0.86 | 0.40 | 1.07 | 4075.45 | 396.22 | 428.04 | 395.24 | 57.80 |
| Nature Safe | 7 | 3.06 | 5.80 | 0.25 | 1.78 | 0.44 | 0.93 | 124.02 | 9.93 | 79.22 | 192.54 | 23.73 |
| Dramatic | 2 | 1.75 | 0.83 | 0.18 | 0.01 | 0.53 | N/A | 11.56 | 2.80 | 1.67 | 15.12 | N/A |

Data provided by the manufacturer and Texas A&M AgriLife Extension Service Soil, Water, and Forage Testing Laboratory (College Station, TX, USA). N/A represents data not available.

**Table S3**. Two-way ANOVA degree of freedom and F value for germination, plant growth, chlorophyll content, and chlorophyll fluorescence parameters.

|  | DF | Germination rate | Emergence index | Shoot FW | Shoot DW | Shoot water content | Root DW | Root water content | SPAD | Fv/Fm | | PI_abs_ |
| --- | --- | --- | --- | --- | --- | --- | --- | --- | --- | --- | --- | --- |
| Fert | 3 | 3.56 | 3.93 | 84.84 | 21.09 | 85.45 | 13.97 | 2.40 | 25.76 | | 14.32 | 2.82 |
| Dose | 3 | 3.02 | 7.28 | 219.80 | 94.96 | 147.11 | 4.18 | 2.34 | 34.07 | | 12.27 | 8.45 |
| F x D | 9 | 1.93 | 2.69 | 3.19 | 1.22 | 6.17 | 5.33 | 2.37 | 2.18 | | 4.07 | 3.76 |

‘Fert’ and ‘F’ refer to the type of fertilizer; ‘Dose’ and ‘D’ refer to the nitrogen dose; ‘DF’ refers to degree of freedom; ‘FW’ refers to fresh weight; ‘DW’ refers to dry weight.

**Table S4**. Two-way ANOVA degree of freedom and F value for shoot morphological and growth parameters.

|  | DF | Hypocotyl length | Plant height | Growth index | Leaf number | Total leaf area | Root to shoot DW | Stem diameter | Compactness |
| --- | --- | --- | --- | --- | --- | --- | --- | --- | --- |
| Fert | 3 | 19.91 | 69.01 | 57.39 | 63.59 | 81.12 | 17.13 | 75.96 | 9.69 |
| Dose | 3 | 21.90 | 162.65 | 159.62 | 148.60 | 215.72 | 15.87 | 74.92 | 0.81 |
| F x D | 9 | 3.01 | 4.19 | 1.58 | 1.49 | 2.62 | 0.48 | 1.50 | 2.64 |

‘Fert’ and ‘F’ refer to the type of fertilizer; ‘Dose’ and ‘D’ refer to the nitrogen dose; ‘DF’ refers to degree of freedom; ‘Root/shoot’ refers to root to shoot dry weight ratio.

**Table S5**. Two-way ANOVA degree of freedom and F value for root morphology parameters.

|  | DF | Total root length | Root area | Average root diameter | Root volume | Number of root tips | Number of root crossings |
| --- | --- | --- | --- | --- | --- | --- | --- |
| Fert | 3 | 87.71 | 92.01 | 18.25 | 84.17 | 50.14 | 66.53 |
| Dose | 3 | 7.64 | 5.61 | 11.35 | 2.93 | 3.26 | 4.70 |
| F x D | 9 | 2.21 | 2.65 | 2.51 | 3.11 | 1.17 | 2.17 |

‘Fert’ and ‘F’ refer to the type of fertilizer; ‘Dose’ and ‘D’ refer to the nitrogen dose; ‘DF’ refers to degree of freedom.

**Table S6**. Mineral contents in substrate under different fertilizer treatments.

| Treatment | | Ca | Mg | S | Na | Fe | Zn | Mn | Cu |
| --- | --- | --- | --- | --- | --- | --- | --- | --- | --- |
| unit | | mg/kg | mg/kg | mg/kg | mg/kg | mg/kg | mg/kg | mg/kg | mg/kg |
| Conventional 1 | | 1000 a | 249 de | 22 bcd | 21 f | 6.96 de | 1.40 c | 1.00 d | 0.74 d |
| Conventional 2 | | 815 ab | 243 e | 23 bcd | 20 f | 6.65 de | 1.42 c | 0.98 d | 0.76 cd |
| Conventional 3 | | 684 bcd | 229 e | 19 bcd | 24 f | 5.37 e | 1.34 c | 1.06 bcd | 0.74 d |
| Conventional 4 | | 776 abc | 250 de | 24 bcd | 19 f | 5.32 e | 1.49 c | 1.02 cd | 0.81 cd |
| Sustane 1 | | 465 d | 266 cde | 8 d | 32 f | 12.67 a | 0.96 d | 1.81 a | 0.62 de |
| Sustane 2 | | 536 bcd | 269 cde | 21 bcd | 43 ef | 13.62 a | 1.58 c | 1.98 a | 0.98 c |
| Sustane 3 | | 571 bcd | 293 cde | 43 b | 61 def | 12.83 a | 2.38 b | 1.89 a | 1.42 b |
| Sustane 4 | | 708 bcd | 356 bc | 102 a | 95 cd | 11.66 ab | 3.22 a | 1.78 a | 1.94 a |
| Nature Safe 1 | | 541 bcd | 323 bcde | 11 d | 39 ef | 11.85 ab | 0.57 e | 1.60 ab | 0.24 g |
| Nature Safe 2 | | 542 bcd | 344 bcd | 6 d | 41 ef | 8.90 bcd | 0.58 e | 1.72 a | 0.21 g |
| Nature Safe 3 | | 517 cd | 409 ab | 15 cd | 48 def | 6.10 de | 0.73 de | 1.57 abc | 0.22 g |
| Nature Safe 4 | | 566 bcd | 454 a | 24 bcd | 58 def | 5.35 e | 0.77 de | 1.52 abcd | 0.25 g |
| Dramatic 1 | | 563 bcd | 300 cde | 7 d | 81 cde | 10.70 abc | 0.55 e | 1.47 abcd | 0.36 fg |
| Dramatic 2 | | 499 cd | 275 cde | 18 cd | 124 c | 7.80 cde | 0.53 e | 1.64 a | 0.36 fg |
| Dramatic 3 | | 565 bcd | 313 bcde | 37 bc | 177 b | 6.34 de | 0.58 e | 1.62 ab | 0.40 fg |
| Dramatic 4 | | 626 bcd | 343 bcd | 80 a | 364 a | 4.77 e | 0.62 e | 1.58 abc | 0.47 ef |
| p | Fert | <0.0001 | <0.0001 | <0.0001 | <0.0001 | <0.0001 | <0.0001 | <0.0001 | <0.0001 |
|  | Dose | 0.1212 | <0.0001 | <0.0001 | <0.0001 | <0.0001 | <0.0001 | 0.4361 | <0.0001 |
|  | Fert x Dose | 0.0141 | 0.0552 | <0.0001 | <0.0001 | 0.0003 | <0.0001 | 0.9622 | <0.0001 |
| F | Fert | 23.35 | 38.38 | 31.29 | 257.04 | 95.75 | 581.33 | 43.74 | 474.37 |
|  | Dose | 2.09 | 11.67 | 71.37 | 75.34 | 32.38 | 98.64 | 0.93 | 64.58 |
|  | Fert x Dose | 2.84 | 2.14 | 16.72 | 39.23 | 5.10 | 66.70 | 0.32 | 43.53 |

‘Fert’ refers to fertilizer type. ‘Dose’ refers to nitrogen dose.

Different letters within one column suggest significant differences among fertilizer treatments indicated by Tukey’s honestly significant difference test at *P* < 0.05.

The last six rows show the two-way ANOVA test results of p-values and F values.

**Table S7**. Mineral contents in leaf of watermelon seedlings under different fertilizer treatments.

| Treatment | | Ca | Mg | S | Na | Fe | Zn | Mn | Cu |
| --- | --- | --- | --- | --- | --- | --- | --- | --- | --- |
| unit | | g/kg | g/kg | g/kg | mg/kg | mg/kg | mg/kg | mg/kg | mg/kg |
| Conventional 1 | | 25.4 abcd | 10.73 bc | 2.64 cde | 308 d | 37.7 cd | 62.6 bcdef | 25.6 e | 10.0 def |
| Conventional 2 | | 25.3 abcd | 11.66 bc | 3.68 bc | 322 d | 61.4 a | 65.8 bc | 34.4 de | 12.1 bc |
| Conventional 3 | | 13.1 cd | 6.67 c | 3.95 ab | 506 d | 61.0 ab | 54.5 cdefg | 25.6 e | 12.7 ab |
| Conventional 4 | | 15.0 bcd | 8.29 c | 3.87 ab | 545 d | 75.3 a | 57.4 cdef | 35.0 de | 14.2 a |
| Sustane 1 | | 32.4 a | 16.21 abc | 2.49 de | 291 d | 37.0 cd | 62.9 bcde | 71.1 ab | 9.3 defg |
| Sustane 2 | | 28.7 abc | 13.55 abc | 2.62 cde | 301 d | 36.8 cd | 64.7 bc | 68.9 ab | 10.2 cdef |
| Sustane 3 | | 29.1 abc | 14.76 abc | 3.96 ab | 444 d | 46.0 bc | 75.5 ab | 74.4 a | 13.2 ab |
| Sustane 4 | | 27.5 abcd | 12.61 bc | 3.59 bc | 470 d | 37.6 cd | 80.2 a | 56.0 abc | 14.7 a |
| Nature Safe 1 | | 22.8 abcd | 14.86 abc | 1.63 e | 212 d | 10.5 fg | 50.1 defg | 47.9 cd | 7.3 g |
| Nature Safe 2 | | 25.1 abcd | 25.74 a | 2.42 de | 265 d | 14.6 efg | 57.9 cdef | 62.7 abc | 8.2 fg |
| Nature Safe 3 | | 17.8 abcd | 15.69 abc | 3.33 bcd | 356 d | 24.4 def | 63.7 bcd | 61.4 abc | 10.5 cde |
| Nature Safe 4 | | 21.0 abcd | 17.97 abc | 3.73 b | 339 d | 28.3 de | 67.7 abc | 73.6 a | 11.3 bcd |
| Dramatic 1 | | 31.5 ab | 23.12 ab | 2.39 de | 634 d | 7.5 g | 49.1 fg | 52.2 bcd | 7.5 g |
| Dramatic 2 | | 16.3 abcd | 14.38 abc | 3.57 bc | 1762 c | 17.4 efg | 41.7 g | 48.4 cd | 9.1 efg |
| Dramatic 3 | | 17.6 abcd | 10.52 bc | 4.11 ab | 2505 b | 20.8 efg | 50.7 defg | 60.7 abc | 11.3 bcd |
| Dramatic 4 | | 11.0 d | 10.50 bc | 4.84 a | 4664 a | 27.3 de | 49.9 efg | 55.7 abc | 10.6 cde |
| Sufficiency range* | | 10-40 | 2.5-10 | 2.5-10 |  | 50-300 | 27-100 | 20-300 | 5-30 |
| p | Fert | 0.0003 | 0.0001 | <0.0001 | <0.0001 | <0.0001 | <0.0001 | <0.0001 | <0.0001 |
|  | Dose | 0.0008 | 0.0190 | <0.0001 | <0.0001 | <0.0001 | 0.0010 | 0.0922 | <0.0001 |
|  | Fert x Dose | 0.1050 | 0.0301 | 0.0150 | <0.0001 | 0.0002 | <0.0001 | 0.0006 | 0.0125 |
| F | Fert | 8.55 | 9.45 | 16.66 | 217.80 | 178.37 | 51.10 | 75.79 | 58.31 |
|  | Dose | 7.14 | 3.83 | 58.72 | 50.06 | 32.50 | 6.95 | 2.34 | 93.25 |
|  | Fert x Dose | 1.81 | 2.45 | 2.81 | 34.63 | 5.27 | 5.97 | 4.64 | 2.90 |

* Data source: Approximate sufficiency ranges of the minerals in mature leaf tissue [1,2].

‘Fert’ refers to fertilizer type. ‘Dose’ refers to nitrogen dose.

Different letters within one column suggest significant differences among fertilizer treatments indicated by Tukey’s honestly significant difference test at *P* < 0.05.

The last six rows show the two-way ANOVA test results of p-values and F values.

References:

1. Kalra, Y. *Handbook of Reference Methods for Plant Analysis*; CRC press, 1997; ISBN 1420049399.

2. Bryson, G.M.; Mills, H.A.; Sasseville, D.N.; Jones, J.B.; Barker, A. V *Plant Analysis Handbook III: A Guide to Sampling, Preparation, Analysis, Interpretation and Use of Results of Agronomic and Horticultural Crop Plant Tissue*; Micro-Macro Publishing, Incorporated, 2014; ISBN 187814801X.
